# Implementation of tuberculosis infection control in health facilities in Mukono and Wakiso districts, Uganda

**DOI:** 10.1186/1471-2334-13-360

**Published:** 2013-08-01

**Authors:** Esther Buregyeya, Fred Nuwaha, Suzanne Verver, Bart Criel, Robert Colebunders, Rhoda Wanyenze, Joan N Kalyango, Achilles Katamba, Ellen MH Mitchell

**Affiliations:** 1 Makerere University College of Health Sciences, School of Public Health, Kampala, Uganda; 2 KNCV Tuberculosis Foundation, The Hague, the Netherlands; 3 Institute of Tropical Medicine, Antwerp, Belgium; 4 University of Antwerp, Antwerp, Belgium; 5 Clinical Epidemiology Unit, College of Health Sciences Makerere University, Kampala, Uganda

**Keywords:** TB infection control, Implementation, Health care workers, Health facilities, Barriers, Uganda

## Abstract

**Background:**

Tuberculosis infection control (TBIC) is rarely implemented in the health facilities in resource limited settings. Understanding the reasons for low level of implementation is critical. The study aim was to assess TBIC practices and barriers to implementation in two districts in Uganda.

**Methods:**

We conducted a cross-sectional study in 51 health facilities in districts of Mukono and Wakiso. The study included: a facility survey, observations of practices and eight focus group discussions with health workers.

**Results:**

*Quantitative*: Only 16 facilities (31%) had a TBIC plan. Five facilities (10%) were screening patients for cough. Two facilities (4%) reported providing masks to patients with cough. Ventilation in the waiting areas was inadequate for TBIC in 43% (22/51) of the facilities. No facility possessed N95 particulate respirators.

*Qualitative*: Barriers that hamper implementation of TBIC elicited included: under-staffing, lack of space for patient separation, lack of funds to purchase masks, and health workers not appreciating the importance of TBIC.

**Conclusion:**

TBIC measures were not implemented in health facilities in the two Ugandan districts where the survey was done. Health system factors like lack of staff, space and funds are barriers to implement TBIC. Effective implementation of TBIC measures occurs when the fundamental health system building blocks -governance and stewardship, financing, infrastructure, procurement and supply chain management are in place and functioning appropriately.

## Background

Interest to eliminate tuberculosis (TB) transmission in health facilities is growing in importance because of the association between TB and HIV and the emergence of multidrug resistant TB (MDR-TB) and extensively drug-resistant TB (XDR-TB) [1]. HIV is a risk factor for developing TB. In addition to reactivating latent *Mycobacterium Tuberculosis* (MTB) infection [2], HIV increases the risk of rapid TB progression after infection or re-infection with MTB [3,4]. Moreover, the risk of TB transmission in health facilities from individuals with TB to other patients and health care workers (HCWs), causing substantial morbidity and mortality is well documented [5-7]. The risk of nosocomial transmission of TB is high in sub-Saharan Africa, where TB and HIV prevalence are high [6,8,9]. This risk is greater when larger numbers of infectious (smear-positive) TB patients are managed at health-care facilities, that don’t have effective infection-control measures [1,10]. The situation is worsened by the increasing number of patients without corresponding infrastructure expansion and health care worker (HCW) recruitment, leading to overcrowding of patients, delayed diagnosis and treatment resulting into increased TB transmission [11]. In addition, challenges such as the impact of dual TB and HIV epidemic and the increasing TB drug resistant cases (M/X/DR-TB) [6,9,12,13] have stimulated the need for strengthening the TB prevention and control in health facilities as well in community settings.

The World Health Organization (WHO) recommends four types of infection control measures in health facilities: managerial measures, administrative measures, proper ventilation and personal protective equipment [14]. These measures have been found to minimize the transmission of TB in health facilities [15,16]. WHO therefore recommends that all health facilities caring for TB patients or persons suspected of having TB implement these measures [1]. Previous research has found that although TBIC guidelines are available for resource-limited settings, their implementation is inadequate [17]. In addition, it has also been found that HCWs often lack knowledge about TB and infection control [18]. Moreover, infection control measures are often not implemented even when HCWs are well informed [19-24]. Lately, the Ministry of Health of Uganda (MOH) and the Tuberculosis Control Assistance Programme (TBCAP) initiated efforts to implement TBIC by training HCWs. However, the extent to which TBIC measures have been implemented in district health facilities has not been evaluated. HIV prevalence in Uganda is high, at 7.3% among adults aged 15–49 years. Uganda ranks 20 among 22 countries with a high burden of TB; 54% of TB patients are HIV co-infected and about 30% of the HIV-related deaths are attributed to TB [25]. In 2010, MDR-TB posed a problem in 1.1% of new cases and 12% of retreatment cases [13]. In addition, Uganda has a case detection rate of 61%, a treatment success rate of 68% and a treatment failure rate of 10% [25]. This calls for an assessment of TBIC measures in health facilities in Uganda. To our knowledge no study has looked at the implementation of TBIC measures in health facilities. Therefore, the purpose of this study was to assess the implementation of TBIC in health facilities in Mukono and Wakiso districts. In addition, we determined whether facility characteristics were associated with implementation of TBIC and identified barriers to implementation.

## Methods

### Study design, population and setting

We conducted a mixed methods cross-sectional study using quantitative and qualitative data collection methods. The study was conducted in two central districts of Mukono and Wakiso in Uganda. These two districts surrounding the capital Kampala are partly semi-urban but largely rural [26]. The HIV prevalence in the central region where Mukono and Wakiso districts are found is the highest in the country, at 12.5% and 8.5% among women and men, respectively [27]. Both districts had a low TB case-detection rate: 46% in Mukono and 38% in Wakiso (MOH, unpublished report, 2008) compared to the WHO TB global target of 70% [25]. However, this has increased to 73% and 68% in Mukono and Wakiso districts, respectively (MOH, unpublished report, 2011). In both districts, training in TBIC was conducted 1–2 years preceding this survey. In an effort to implement TBIC measures, training of HCWs in TBIC was carried out by MOH and TBCAP in Wakiso and Mukono districts, respectively. Details of the trainings have been described elsewhere [28]. Health services in Uganda are provided by the government, private-not-for-profit (PNFP), and privately owned health facilities. The government health system in Uganda is decentralized at the district level. It is arranged in a hierarchy starting with village health committees which act as an outpost for outreach services at the village level, health centre (HC) II at a parish level (serving about 5,000 people), HCIII at a sub-county level (serving about 25,000 people), HCIV at the sub-district level (about 100,000 people) and the District Hospital [29]. Each level offers what is at the lower level plus additional services for its own level. TB services are available at HCIIIs and above. For the purpose of this study, only government and PNFP health facilities were included in the study (except those located on islands). The study was conducted prior to release of the Ugandan National Policy on TBIC [30].

### Data collection

Data collection was carried out from October 2010 to February 2011. A facility survey, participant observations, review of facility records, and focus group discussions (FGDs) with HCWs were conducted. The FGDs were meant to elicit in-depth understanding of those factors influencing implementation of TBIC measures.

#### Quantitative data

##### Health facility survey

A facility survey was conducted in all public and PNFP facilities from hospital to HCIII level. The survey assessed whether TBIC measures were being implemented. This was assessed through interviews of the facility managers and in their absence, the TB/HIV focal persons or the officer in-charge of the TB clinic and by observation of TBIC practices. The facility questionnaire sought information on the characteristics of the facility (facility level and ownership), general and TB patient load and the TBIC measures available based on the recommended WHO 2009 guidelines for the prevention of TB in health care facilities [1]. These included availability of a TBIC plan, HCWs training, prompt identification of TB suspects, triage, patient education about TB, sputum collection practices (in a well ventilated area), well ventilated facilities and use of protective wear. This facility instrument was adopted from a standardized tool for assessment of adherence to recommended TBIC from the manual for implementing TB control measures in health care facilities [31].

In addition, unannounced direct observations of control measures were carried out in order to have a more objective assessment as recommended [32]. These observations were conducted on three weekdays for each health facility. Using a checklist, data were collected on patient screening for cough, where the screening was done, availability of masks for patients with cough, whether the TB suspects had a separate waiting area, and triaging of TB suspects in the outpatient department. The observations were recorded as ‘always’ if the practice was observed for the three days of observation, ‘occasionally’ if the practice was not consistently observed, or ‘not at all’ if the practice was not seen. These observations were used to derive an average/overall score for the respective health facilities. Observed HCWs were not aware that they were being observed, as this would have created a bias. These observations provided convergent validity to the responses from the managers.

We also collected data on space and ventilation adequacy at the waiting areas, consultation rooms, in-patient wards and laboratories. Appropriateness of air ventilation was assessed using the ratio of the window area to the floor area/room area as calculated in the formula below, with the cut off being ≥ 20%. This was adapted from the tool described in the appendix for the Uganda National TBIC guidelines [30].

Area room (ARM) = Length *Width= M^2^

Area window opening (ARW);

Window (W) = Area (Width*Height)

Total window area in a room (ARW) = W1+ W2+…=..W^2^

Then, ARW/ARM × 100% ≥ 20% (the acceptable ventilation)

#### Qualitative data

FGDs with the use of an interview guide explored HCWs’ experiences and perceived challenges in implementing the TBIC measures. Eight FGDs were conducted with HCWs. Seven FGDs with female HCWs (working in outpatient departments, HIV clinics, medical wards and TB clinics) and one with males (including laboratory personnel and clinical officers) were conducted. The same FGDs were used for assessing utilization of HIV and TB services by HCWs in Uganda. Some of these questions were sensitive and so we found it necessary to stratify HCWs by gender. The detailed methods for these FGDs have been described elsewhere [28].

#### Quality control

The study tools (questionnaire, FGD guide and checklist) were pretested and revised accordingly. Experienced research assistants were trained on the objectives of the study and how to administer the tools. Both HCW and facility interviews were undertaken in private rooms offering confidentiality. Quantitative data were double entered into a computer.

#### Data management and analysis

Data from the health facility survey were entered in Epi-Info version 3.2.2 software and then cleaned before being exported to STATA version 10 for analysis.

##### Health facility survey

The facility assessment included both data from the interviews and the observations. Data from the observations was further collapsed into ‘always practiced’ for those that were initially categorized as ‘always’ and ‘occasionally practiced’ and then ‘not at all’ for those which were not implementing the practice. Chi-square tests and multivariate logistic regression were used to assess factors associated with two of the TBIC measures i.e.; availability of TBIC plan and screening for TB suspects (cough lasting 2 weeks and above) as they arrive at the health facility. These were chosen as they represent managerial and administrative level measures of TBIC [1]. Fishers Exact test was used to analyze associations for categorical data. The level of significance was set at 5%.

The FGDs were transcribed. Transcripts were read several times to get an overall picture of the content. Data was analyzed manually and common themes developed. Members of the research team reviewed themes for agreement. The qualitative findings complemented findings from the facility survey (questionnaire and observation) data.

#### Ethical considerations

The study was approved by the Makerere University School of Public Health Higher Degrees Research and Ethics Committee and the Uganda National Council for Science and Technology (Ref.nr. HS 880). Informed written and verbal consent was obtained for the surveys and FGDs, respectively, from participants at the time of data collection. Confidentiality and use of data for research purposes was emphasized prior to the beginning of the data collection.

## Results

More than half (65%) of the facilities were HCIIIs. Most facilities (80%) were government owned, Table 1. The median annual patient load/patient encounters in outpatient clinics was 16,212 (IQR 11,500-22,496). The median number of TB patients seen/diagnosed in the facilities annually was 24. Almost a quarter of the facilities did not have TB diagnostic facilities on site.

**Table 1:** General characteristics of health facilities in Mukono and Wakiso districts

**Variable**	**n=51**	**%**
**Number of facilities**		
Mukono district	21	41.2
Wakiso district	30	58.8
**Facility level**		
Hospital	10	19.6
HC IV	8	15.7
HC III	33	64.7
**Facility ownership**		
Government	41	80.4
Private-Not-For-Profit	10	19.6
**Outpatient turnover per year**		
Median	16,212 (IQR 11,500-22,496)	-
**TB patient turnover per year**		
Median	24 (IQR 12–61)	-
**Staffing at health facilities**		
Median	12 (IQR10-25)	-
**TB diagnosis**		
On-site	39	76.5
Off-site (requires referral)	12	23.5

HC=Health Centre.

IQR= Interquartile range.

### TB infection control measures in the facilities

#### Facility level managerial measures

##### Availability of TBIC plan

Only 16/51 (31%) facilities had a TB infection control plan. Facilities which reported training their staff in TBIC were more likely to have a TBIC plan. However, facility level and ownership were not associated with having a TBIC plan, (Table 2). In a multivariable model, facilities from Mukono district were more likely to have TBIC plan than those of Wakiso (adjusted Odds Ratio [aOR] = 34.9 (5.9-204.7).

**Table 2:** Facility characteristics associated with availability of a TB infection plan in health facilities in Mukono and Wakiso districts

**Characteristics**	**Availability of TBIC plan**	**Crude OR (95% CI)**	**Adjusted OR**	***p*-value**
**District**				
Wakiso	2/30 (6.7%)	1	1	
Mukono	15/21 (71.0%)	35 (6.3-195.2)	34.9 (5.9-204.7)	<0.01
Facility level*				
Primary	9/33 (27.3%)	1	1	
Tertiary	8/18 (44.4%)	2.1 (0.64-7.1)	2.1 (0.25-18.5)	0.49
**Ownership**				
PNFP	5/10 (50%)	1	1	
Government	12/41 (29.3%)	0.41 (0.101-1.7)	1.2 (0.91-14.8)	0.91
**Staff training in TB IC**				
No	0/16 (0%)			
Yes	17/35 (48.6%)			
**Staff numbers (quartiles)**				
>9	2/10 (20.0%)	1	-	-
10-11	1/11 (9.1%)	0.4 (0.03-5.24)		
12-24	3/12 (25.0%)	1.3 (0.17-10.1)		
25-118	7/13 (53.9%)	4.7 (0.70-31.0)		

*Primary facilities consist of HCIIIs and tertiary level includes HCIVs and hospital levels.

#### Administrative measures

##### Screening of TB suspects

At the outpatient’s departments, less than half of the facilities (22/51; 43%) reported screening patients for cough as they enter the facility, Table 3. However, direct observation showed that 46/51 (90%) of the facilities did not screen patients at all, only three facilities occasionally screened while two facilities always screened patients. Facilities from Mukono districts were more likely to report screening patients for cough than those from Wakiso (OR=2.7; 95% CI= 0.84-8.42), although the difference was not statistically significant. Facilities with high patient load were more likely to screen than those with few patients; (Table 4). Facility level, ownership, staff numbers in the facility and report of staff training in TBIC were not associated with observed patient screening. In a multivariable model, facilities from Mukono districts were more likely to report screening of patients for cough (aOR= 6.2; 95% CI= 1.10-34.58). No factors were associated with observed (always and occasionally) patient screening for cough in outpatient departments.

**Table 3:** Observed and reported administrative measures in facilities enhancing TB infection control in Mukono and Wakiso districts

**TB infection control measures**		**Observations**		**Interviews**		***p*-value**
		**n**	**%**	**n**	**%**	
1. Patients screened for cough as they enter the facility	Yes	5	9.8	22	43.1	0.08
2. TB suspects fast tracked (triaged)	Yes	4	7.8	25	49	0.03*
3. TB suspects separated from other patients	Yes	5	10	28	55	0.03*
4. TB suspects provided with masks	Yes	0		2	96	-
5. Designated & well ventilated area for sputum collection	Yes	-	-	16/38	42	-

*Observed practices were statistically different from the reported.

**Table 4:** Facility characteristics associated with reported patient screening for cough in Mukono and Wakiso health facilities

**Characteristics**	**Patient screening**	**Crude OR (95% CI)**	**Adjusted OR (95% CI)**	***p*-value**
**District**				
Wakiso	10/30 (33.3%)	1	1	
Mukono	12/21 (57.1%)	2.7 (0.84-8.42)	6.2 (1.10-34.58)	0.04
**Facility level**				
Primary	12/33 (36.4%)	1	1	
Tertiary	10/18 (55.6%)	2.2 (0.68-7.0)	0.2 (0.01-2.70)	0.21
**Ownership**				
PNFP	5/10 (50.0%)	1		
Government	17/41 (41.5%)	0.71 (0.17-2.83)	-	-
**Staff training in TB IC**				
No	0/16 (0.0%)	-		
Yes	22/35 (62.9%)			
**Staff numbers (quartiles)**				
>9	3/10 (30.0%)	1	1	
10-11	2/11 (18.2%)	0.5 (0.7-4.0)	1.0 (0.92-11.28)	0.92
12-24	8/12 (66.7%)	4.7 (0.8-28.5)	4.9 (0.37-64.33)	0.23
25-118	8/13 (61.5%)	3.7 (0.6-21.6)	7.80 (0.27-225-78)	0.23
**Annual outpatient turnover (quartiles)**				
<8499	1/11 (9.1%)	1	1	
8,500-17,999	5/11 (45.5%)	8.3 (0.78-89.5)	4.2 (0.29-59.98)	0.29
18,000-66,442	6/11 (54.5%)	12 (1.1-128.8)	9.5 (0.70-127.89)	0.09
>66,443	8/12 (66.7%)	20 (1.85-216.2)	19.6 (1.05-366.79)	0.05

##### Other administrative measures

Coughing patients were not given priority in outpatients departments in over 90% (47/51) of the facilities (Table 3). They were observed waiting in the same area with other patients for long hours in queues. Only two facilities reported providing masks/tissues to coughing patients, with one facility observed giving them only to confirmed TB patients.

#### Environmental measures at the waiting area and consultation rooms

Ventilation measurents were only available for 50 health facilities. Almost a half (22/50) of the facilities didn’t have adequate ventilated waiting areas based on the proportion of the window to floor area, Table 5. In addition, only 32% (17/50) of the facilities had the available windows fully open. In two facilities, there were louvered windows, with a wire mesh on the outside part of the windows, which prevented complete opening of the louvers, thus compromising ventilation. Structural improvements were reported in only three facilities and these facilities had a tent as waiting area. In most facilities, patients were observed to crowd in narrow and poorly ventilated corridors in outpatient departments, even where there was open area to wait from. Twenty four percent (12/51) of the facilities were not providing sputum diagnosis on site. For the facilities that were carrying out sputum diagnosis on site, 22/38 (58%) reported not having a designated area away from other patients and staff where patients can produce sputum specimens. Of those facilities which reported a designated area for sputum collection, 27% (4/16) reported this area being in the open air, 40% (6/16) near or behind the toilet, and 33% (6/16) anywhere in the facility.

**Table 5:** **Ventilation assessment of out**- **and inpatient departments**, **consultation rooms**, **and laboratories in health facilities in Mukono and Wakiso**

**Area assessed Number examined**		**Number with adequate, n =50**	**%**
Outpatient Department	Adequate	28	56
	Not adequate	22	44
Consultation rooms	Adequate	22	44
	Not adequate	28	56
In-patient ward*	Adequate	17	37
	Not adequate	29	63
Laboratory*	Adequate	17	37
	Not adequate	29	63

*Some facilities didn’t have in-patient and laboratory facilities.

Adequate ventilation if ARW≥20% of ARM.

### Personal protective equipment

No facility had N95 respirators. In one facility, we observed sputum induction being carried on the ward with HCWs only wearing surgical masks to protect themselves.

### Perceived barriers to implementation of TB infection control

#### Qualitative results

##### Structural barriers

A number of challenges were raised by HCWs. The most commonly cited barriers were; lack of space to implement the separation of TB suspects, both out and in-patient settings. Although the HCWs knew that TB suspects should be separated, it was reported that this was not possible due to lack of space in most facilities.

‘*It is not possible here* [*patient separation*] *because we don*’*t have enough space*. *We don*’*t have a place where to put them* [*TB suspects*].’ FGD Females

It was also reported in some FGDs that waiting rooms in facilities were small and poorly ventilated. All FGDs reported that, even minor structural changes were not possible. However, it was mentioned by three FGDs that because of the crowded and poor ventilated waiting areas, the TBCAP project gave them tents to be used as waiting areas. Unfortunately, in two facilities out of the three that were given tents, it was reported that the tents got torn. There was also a problem of not having chairs for the tents.

##### Lack of human resources

All FGDs reported that understaffing in their health facilities was a problem. TBIC measures like screening for people with cough, health education and timely sputum examination were seen as additional tasks for the already overstretched staff. It was reported that screening for cough as patients arrive was not done. In facilities where screening was done; it was reported to be carried out only in the consultation room and not as soon as they arrive at the facility.

‘*Because sometimes you are busy doing other things you may not have time*; *you are busy giving injections*. *We are not so many*; *like now I am alone*; *I am the only enrolled nurse*. *If we were at least two*, *we would be doing the screening as they* [*patients*] *come*… *You just hear somebody coughing when you are here in the room*, *when you get out*, *you can*’*t even know who has been coughing*. *May be you ask and it may look embarrassing to a patient*; *they might say why are they asking me*, *is it bad to cough*? *So*, *it needs somebody who can sit there to just observe them*.’ FGD Females

In one facility, it was reported that expert clients (HIV patients who work as volunteers) were used to give health education to patients in the waiting area, as a way to reduce the workload for the HCWs.

##### Stigma attached to TB

It was mentioned that there was reluctance of the staff to screen for patients with cough and separating them from other patients. Some FGDs reported that they were hesitant to tell a patient that they were suspecting TB and separating them from other patients when the TB diagnosis was not confirmed. Others felt that asking for cough at the reception (as the patient arrives) is part of history taking which should be done in the consultation room and not in public like in the waiting area. TB suspects were left seated with the other patients until it was their turn to go (‘first come first serve’) to the consultation room where screening for cough took place.

‘*The problem is that we find it hard to tell them that*, *we are suspecting this* [*TB*], *you are not supposed to be with other people*, *sit alone*. *After all*, *the results might turn out to be negative*.’ FGD men

##### Managerial support

Lack of funds to buy masks and to carry out renovations and structural improvements were also reported as challenges.

‘…*But they didn*’*t carry it out*; *we put in our action plan to put some aeration to allow air flow through some of the corridors but it wasn*’*t done because of the funds*.’ FGD Women

In one facility it was reported that the administration was not supportive enough, even when health workers made a request to shift the HIV clinic in their facility from a poorly ventilated place to an outdoor one.

“*Like you see our TB*/*HIV premises*, *we are at a higher risk and when we were conducting the TB infection assessment*, *the ventilation was found to be at 0*%. *There is even darkness in the area and the worst thing is that these HIV patients are seated together with TB patients and some of them are suspects*, *and not yet on treatment*. *We requested to move the clinic to a more open*, *better ventilated place out there on the verandah*, *but it was rejected*. *So TB CAP came in and gave us a tent*, *which tent didn*’*t help us much because it came in without seats and immediately we put it up*, *it broke down and we didn*’*t use it*. *So we have remained in the same place up to today*.” FGD Females.

##### Negative attitude towards TB among health care workers

Some FGDs reported that some HCWs have negative attitudes towards TB work. It was expressed that many HCWs were not interested in TB related work and therefore TBIC control is left to the staff working in the TB clinics. Some felt that there is no need to worry, since they have previously worked in the same environment without getting TB.

“*Some clinicians don*’*t comply with separating TB suspects from other patients*. *They don*’*t appreciate why TB suspects should be triaged.*” FGD male HCWs

“*Am just wondering that recently they are making it a serious issue that health workers are at risk of acquiring TB from patients*. *We have worked here for years treating TB patients and none of us has ever got TB*. *Why the fuss now*?” FGD Female HCWs

Two FGDs mentioned that support supervision and encouragement by district health officials to implement TB IC would motivate HCWs.

##### Lack of adherence among patients

The other challenges mentioned included patients being non-compliant with cough hygiene and not accepting to be separated. It was reported that where HCWs make an effort to implement the TBIC measures, patients don’t adhere to the instructions they are given.

## Discussion

The findings in this study provide important information on the current status of implementation of TBIC measures in Uganda. Few health units had a TBIC plan and there was poor implementation of administrative measures like screening and separation of coughers. In addition, almost half of the facilities had poorly ventilated waiting areas. Reported barriers to implementation of TBIC included limited human resource, poor attitude towards TBIC by HCWs, lack of space and lack of funds.

Results from this study show that administrative control measures, the most important and most feasible measures in resource limited setting [1] were not implemented. The situation was worsened by the inadequate ventilation (in waiting areas, consultation rooms and in-patient wards) and failure to open the windows. Reasons like lack of space and poor staffing levels accompanied with heavy workload, being given for the poor implementation of the administrative measures. Similar barriers have been reported in other settings [32,33]. This study has demonstrated a discrepancy between self-reported and observed TBIC practices. While 43% and 55% of the facilities were reported to screen and separate TB suspects, respectively, only 10% of them, were observed implementing both measures. This illustrates that HCWs relatively know what they are expected to do in terms of TBIC, but probably because of poor staffing levels and failure to appreciate the importance of TBIC, recommended measures are not implemented. This discrepancy between reported and observed measures also highlights the weakness of self-reports. Overestimation regarding compliance with guidelines due to socially desirable behavior in self-reports has been cited [34].

The factors reported to hinder implementation of TBIC measures from the qualitative findings, emphasize the need for multi-pronged interventions in order to cause lasting behaviour change [35]. In this study, such interventions would include solutions for human resource constraints, funding, supplies, structural changes in the facilities and managerial support. Thus for effective implementation of TBIC, the six health system building blocks-governance and leadership, health financing, access to supplies and human resource [36] are in critical.

Most importantly, two FGDs mentioned that support supervision would motivate them to practice the TBIC measures. Providing feedback and the awareness of being observed has been reported to enhance hand hygiene [35,37]. This could be utilized in the implementation of TBIC too. Support from the administration in the staff efforts to implement TBIC is necessary for its successful implementation [1]. Institutional administration support has been pointed as critical in general infection control [37].

A qualitative study in Russia, [38] found that fear of contracting TB was a motivator for implementing TBIC measures. In the current study HCWs seemed reluctant in implementing these measures, because they didn’t appreciate the importance of TBIC, after previously working in the same environment, without getting TB. An introduction of surveillance system for TB among HCWs is an important indicator of the quality of TBIC [25]. This is critical considering the fact that Uganda has one of the highest TB default rate in the world of 10% [25], high HIV prevalence of 7.3% [27], the poor implementation of TBIC measures is a cause for concern.

This current study findings show that Mukono district was doing better than Wakiso, possibly due to the difference in the implementation approach. The TBCAP project provided additional support such as tents in some facilities, which was not the case in the MOH supported district. In addition whereas all facilities in Mukono had some people trained only selected facilities in Wakiso had people trained. Therefore, this difference might be due to more comprehensive support in addition to training, and the more inclusive training.

Our study had limitations. We only assessed the availability of IC measures and were unable to differentiate quality, comprehensiveness or consistency of implementation of all measures at assessed sites. For example a patient flow evaluation as an index of effective triage was not carried out. We also did not objectively measure the ventilation of the facilities using smoke tubes and anemometers. Thus we were did not get the actual air exchanges per second. However, the method we used gives an idea on the status of the ventilation. Non-differential misclassification with regard to presence or absence of different IC measures was reduced by use of interviews and observations. Though the tool that was used for the facility survey was not validated, it was pretested and the necessary adjustments made. The study was only carried out in two semi-urban districts in Uganda, which are close to the capital city, Kampala. However, the challenges affecting TBIC implementation i.e. health system challenges are generally the same across the country. Thus the practice and the challenges to implement TBIC in other areas may be even worse than the ones from the study districts. The qualitative findings from the FGDs, though not generalizable, together with the quantitative data provided a better understanding of the findings than either approach alone [39].

## Conclusions

Implementation of TBIC in Mukono and Wakiso districts in Uganda was poor. Limited resources such as lack of staff, funds and space and failure to appreciate the importance of TBIC precluded the adoption of even simple, cheap and most important TBIC interventions. Therefore, in ensuring implementation of the recommended TBIC practices, comprehensive support beyond training (e.g. human resources, providing masks, space and other alterations, support supervision, hands on support in development of TBIC plans and operationalizing them) are important.

## Competing interests

The authors declare that they have no competing interests.

## Authors’ contributions

EB, SV, BC, FN, RC, EMH, AK were involved in the development of the proposal. EB carried out field work. EB, FN, KJN, EMH, RW and RC undertook data analysis. EB, FN, KJN, EMH, SV, AK, RC, RW and BC drafted the manuscript and approved the final draft.

## Pre-publication history

The pre-publication history for this paper can be accessed here:

http://www.biomedcentral.com/1471-2334/13/360/prepub
